# Analytical Performance and Evaluation in Clinical Cohorts of a Fully Automated Immunoassay for Plasma Glial Fibrillary Acidic Protein

**DOI:** 10.3390/diagnostics16132060

**Published:** 2026-07-01

**Authors:** Ben Schlichtmann, Burak Arslan, Kara Johnson, Jason Patzlaff, Dusten Unruh, Miklos Szabo, Mark Holland, Jeff Todtleben, Mike Salvati, Kubra Tan, Ulf Andreasson, Jeremiah Hinson, Scott Levin, Henrik Zetterberg, Andrea Lessa Benedet, Holly Ann Hill

**Affiliations:** 1 Beckman Coulter Diagnostics, 222 Lake Hazeltine Dr, Chaska, MN 55318, USA; bschlichtmann@beckman.com (B.S.); kcurtis01@beckman.com (K.J.); jason.patzlaff@bio-techne.com (J.P.); dunruh@beckman.com (D.U.); mszabo@beckman.com (M.S.); mdholland@beckman.com (M.H.); jtodtleben@beckman.com (J.T.); msalvati@beckman.com (M.S.); 2 Department of Psychiatry and Neurochemistry, Institute of Neuroscience and Physiology, The Sahlgrenska Academy, University of Gothenburg, 40530 Mölndal, Sweden; burak.arslan@gu.se (B.A.); kubra.tan@gu.se (K.T.); ulf.andreasson@gu.se (U.A.); henrik.zetterberg@clinchem.gu.se (H.Z.); andrea.benedet@gu.se (A.L.B.); 3 Danaher Diagnostics, 2200 Pennsylvania Ave., NW Suite 800W, Washington, DC 20037, USA; jeremiah.hinson@dhdiagnostics.com (J.H.); scott.levin@dhdiagnostics.com (S.L.); 4 Department of Emergency Medicine, School of Medicine, Johns Hopkins University, 733 N Broadway, Baltimore, MD 21287, USA; 5 Clinical Neurochemistry Laboratory, Sahlgrenska University Hospital, 41345 Mölndal, Sweden; 6 Department of Pathology and Laboratory Medicine, University of Wisconsin School of Medicine and Public Health, Madison, WI 53705, USA; 7 Wisconsin Alzheimer’s Disease Research Center, University of Wisconsin School of Medicine and Public Health, University of Wisconsin-Madison, Madison, WI 53715, USA; 8 Department of Neurodegenerative Disease, University College London Institute of Neurology, Queen Square, London WC1N 3BG, UK; 9 UK Dementia Research Institute, University College London, London WC1N 3AR, UK; 10 Centre for Brain Research, Indian Institute of Science, Bangalore 560012, India

**Keywords:** glial fibrillary acidic protein, GFAP protein, human, immunoassay, Alzheimer’s disease

## Abstract

**Background/Objectives**: This study aimed to perform analytical validation and evaluation of the clinical performance of the Access Glial Fibrillary Acidic Protein (GFAP) research-use-only (RUO) immunoassay (Beckman Coulter) in plasma. **Methods**: A fully automated GFAP immunoassay was developed and evaluated for analytical validity and clinical performance. Analytical validation assessed precision, sensitivity, linearity, analytical specificity, and the stability of calibrators and samples. Evaluation of predefined clinical performance criteria included method comparison against the Quanterix Simoa GFAP Advantage Plus (RUO) assay and assessment of GFAP levels between participants with Alzheimer’s disease (AD) and healthy controls. **Results**: The Access GFAP (RUO) immunoassay met all analytical criteria. Precision yielded coefficients of variation (CVs) < 10% across all concentration ranges. Sensitivity parameters included a lower limit of quantification (LLoQ) of 0.083 pg/mL and an analytical measurement range of 0.083–640 pg/mL. Linearity demonstrated <9% deviation across the measurement range. Interference testing showed <7% deviation for cross-reactants, common medications and AD-specific therapeutics. Plasma samples remained stable over 48 h at room temperature (<4% deviation) and five freeze–thaw cycles (<9% deviation). Method comparison demonstrated strong correlation with Simoa GFAP (R = 0.945) but systematic proportional bias, yielding 3% (slope = 0.030) of Simoa values. Samples from participants with AD exhibited significantly elevated GFAP levels (median 9.0 pg/mL, IQR: 5.3–19.1) versus controls (median 3.4 pg/mL, IQR: 1.5–6.5; *p* < 0.001). **Conclusions**: The high-throughput Access GFAP (RUO) immunoassay achieved all analytical performance criteria and demonstrated differences in GFAP levels between AD and healthy control sample cohorts. These findings support its use in research settings and as a foundation for future clinical implementation and may inform future studies evaluating cross-platform harmonization and potential clinical applications.

## 1. Introduction

Alzheimer’s disease (AD) is a progressive neurodegenerative disorder that begins decades before symptom onset [1,2]. While amyloid-β (Aβ) and tau serve as molecular hallmarks of AD, recent evidence implicates neuroinflammatory and glial processes in AD initiation and progression. Astrocytic activation represents an early event that may precede detectable neurodegeneration and cognitive decline, making astrocyte biomarkers valuable for early detection, risk stratification, and therapeutic monitoring in preclinical and early-stage AD [3].

Astrocytes play a significant role in gliosis, the reactive response of the central nervous system to injury or disease. This reactive state is characterized by upregulation of glial fibrillary acidic protein (GFAP), an intermediate filament protein unique to astrocytes. Prior studies of traumatic brain injury (TBI) and stroke consistently showed elevated GFAP levels in peripheral blood, reinforcing its role as a sensitive CNS pathology indicator [4]. Although astrogliosis mechanisms in AD remain incompletely understood, evidence suggests glial scarring occurs in regions in which amyloid plaques develop, potentially reflecting responses to Aβ peptides [5,6,7]. Activated astrocytes may release pro-inflammatory cytokines and chemokines, contributing to neurotoxicity and disease progression [8].

GFAP demonstrates particular promise as an early AD biomarker, associated with Aβ deposition, tau pathology in Aβ-positive individuals, and cognitive decline [6,9,10,11,12,13,14,15]. Beyond AD, GFAP shows broader clinical utility with established prognostic value in multiple sclerosis (MS) [16,17] and amyotrophic lateral sclerosis (ALS) [16,18]. Importantly, GFAP concentrations vary substantially by condition—TBI produces levels that far exceed those in AD [4,19]—necessitating pathology-specific interpretation. The advent of ultrasensitive immunoassays has enabled reliable and practical blood-based GFAP measurement, facilitating its application across diverse neurological conditions [20].

These methods for measuring low-abundant proteins have significantly enhanced our understanding of GFAP levels in the serum or plasma of patients with diverse neurological diseases, enabling the application of GFAP as both a diagnostic and a prognostic biomarker across Alzheimer’s disease, traumatic brain injury, multiple sclerosis, and other CNS conditions [20]. Subsequent platform development has focused on translating this analytical capability into high-throughput, fully automated formats compatible with clinical laboratory infrastructure, including chemiluminescent immunoassay analyzers, electrochemiluminescence platforms, and paramagnetic particle-based systems. A critical challenge accompanying this expansion is assay harmonization, as the epitopes targeted by commercially available GFAP immunoassays are mostly unknown or poorly characterized, and, despite measuring the same analyte, available platforms demonstrate substantial inter-platform differences in absolute concentrations driven by differences in antibody clone selection, epitope recognition, calibration strategy, and signal detection mechanisms [20,21]. The absence of an internationally recognized reference material or reference measurement procedure for plasma GFAP means that values generated by different platforms cannot be directly compared without empirical conversion factors, limiting the generalizability of published reference intervals and clinical thresholds across assay systems. Establishing the analytical performance characteristics of each platform independently and characterizing inter-platform relationships are essential prerequisites for clinical implementation.

The objective of this study was to evaluate the analytical validity and clinical performance of the Access GFAP research-use-only (RUO) immunoassay (Beckman Coulter, Brea, CA) in plasma. Analytical validation followed Clinical and Laboratory Standards Institute (CLSI) guidelines [22]. Evaluation of clinical performance included method comparison with the Quanterix Simoa GFAP assay and assessment of GFAP levels in AD participants versus healthy controls.

## 2. Materials and Methods

### 2.1. GFAP Immunoassay

**Figure 1:**
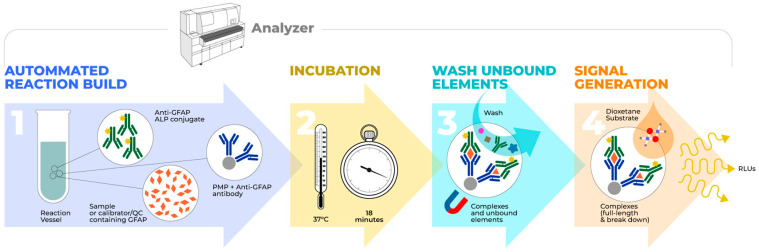
Steps of the Dxl 9000 Access Immunoassay Analyzer.

The Access GFAP (RUO) immunoassay employs an immunoenzymatic sandwich format utilizing paramagnetic particle separation (Figure 1). The Dxl 9000 Access Immunoassay Analyzer (Beckman Coulter, Brea, CA, USA) was employed for precision, sensitivity, linearity, analytical specificity and stability studies. The Access2 Immunoassay Analyzer (Beckman Coulter, Brea, CA, USA) was used for method comparison and clinical performance studies.

The automated assay proceeds through four sequential steps: (1) reaction build, (2) incubation, (3) washing unbound elements, and (4) signal generation and quantification. During reaction build, the analyzer adds 100 µL of sample to a reaction vessel, which is incubated with an ancillary solution. Then, paramagnetic particles coated with anti-GFAP mouse monoclonal capture antibody and alkaline phosphatase-conjugated anti-GFAP mouse monoclonal detection antibody are added to the reaction mixture. The mixture incubates at 37 °C for 18 min, allowing formation of antibody–GFAP–antibody sandwich complexes on the particle surface. Following incubation, a magnetic field immobilizes particle-bound complexes, while washing removes unbound materials. Lastly, a chemiluminescent substrate (dioxetane) is added to the reaction vessel, which, when dephosphorylated by the bound alkaline phosphatase, generates light emission directly proportional to GFAP concentration. The analyzer automatically calculates GFAP levels by comparing signal intensity to a stored 6-point calibration curve spanning 0–640 pg/mL.

From an environmental sustainability perspective, the Access GFAP (RUO) assay demonstrates several attributes consistent with green chemistry principles [23]. The fully automated Dxl 9000 platform requires only 100 µL of plasma per determination, eliminates organic solvents, minimizes analyst handling and associated chemical exposure, and generates less per-test waste than conventional manual ELISA formats. High-throughput operation (450 tests/h) further reduces energy consumption relative to semi-automated platforms, supporting more-resource-efficient deployment in clinical laboratory settings.

### 2.2. Samples and Participants

AD samples used for clinical performance evaluation were obtained from BioIVT (Westbury, NY, USA) and PrecisionMed, LLC (Carlsbad, CA, USA), from participants with clinically diagnosed AD as determined by treating practitioners. Inclusion criteria were clinical diagnosis of AD with mild-to-moderate cognitive impairment (MMSE 16–26), age 50–90 years, and availability of K2 EDTA plasma collected within 24 h of processing. Exclusion criteria included: concurrent neurological diagnoses other than AD or significant systemic comorbidities (chronic kidney disease, diabetes, cardiovascular disease). Samples with gross hemolysis or lipemia were excluded from all analyses.

Age-matched healthy control plasma was obtained in bulk quantities (>250 mL) from three biospecimen repositories: BioIVT (Westbury, NY, USA), Cantor BioConnect, LLC (Santee, CA, USA), and Medical Research Networx, LLC (Franklin, MA, USA). All healthy control plasma samples from participants were confirmed negative for any neurodegenerative diseases and for significant comorbidities (e.g., chronic kidney disease, diabetes, cardiovascular disease).

A small subset of samples (*n* = 8) were obtained from the Clinical Chemistry Laboratory at Sahlgrenska University Hospital (Mönldal, Sweden) and used only for comparative sample stability analyses. These samples were collected without specific criteria or clinical information and are not included in the clinical cohort description.

Samples were stored at −80 °C and thawed overnight at 4 °C before testing. All analytical validity measures were evaluated using plasma samples from healthy controls, except for sensitivity studies, for which samples from participants with AD were also included. Samples with gross hemolysis or lipemia were excluded from all analyses. The final clinical sample cohort comprised AD participants (*N* = 15, median age 69 years, range 52–83, MMSE 16–26) and healthy controls (*N* = 20, median age 65 years, range 55–70).

The study was conducted in accordance with the Declaration of Helsinki. All samples were obtained under institutional review board-approved protocols at each source institution, under which participants provided written or oral informed consent.

### 2.3. Precision

The precision of the GFAP immunoassay was evaluated per CLSI EP05-A3 [24] using native and recombinant GFAP-spiked K2 EDTA plasma samples across the analytical measurement range (AMR). Experiments were conducted over 5 days (3 replicates per run, 2 runs per day). Within-run, between-run, and within-laboratory coefficients of variation (CVs) were calculated for each sample.

### 2.4. Sensitivity

The lower limit of quantification (LLoQ) was evaluated using three native plasma samples (patient pools, which were diluted up to 128× using calibrator matrix) and run in 5 replicates. The LLOQ was evaluated based on the definition in Equation (13) in CLSI guidelines EP17-A2:2012 [25], where the accuracy goal is the extended uncertainty (k = 2) on the CV ratio (CVr) of the pooled sample (CVr = 3.2%) for the plasma pool in the precision calculations. The LLOQ was determined as the point at which the fitted precision profile of the pooled patient sample CV crossed the threshold of k × CVr = 6.4%.

### 2.5. Linearity

Assay linearity was evaluated per CLSI EP06-ED2 [26] across the full AMR. A low-concentration sample (healthy control K2 EDTA) and a high-concentration sample (healthy control K2 EDTA plasma sample spiked with recombinant GFAP antigen) served as endpoints. Seven intermediate samples were prepared by mixing increasing proportions of the high sample with the low sample to span the assay range. All samples were tested, with the low sample measured in 8 replicates and the remaining 8 samples measured in 4 replicates.

To assess linearity with native antigen, a low-concentration sample (calibrator matrix) and a high-concentration sample (healthy control K2 EDTA plasma sample spiked 1:10 with a cerebrospinal fluid pool) served as endpoints. Four intermediate samples were prepared by mixing increasing proportions of the high sample with the low sample to span the low (AD-relevant) range of the assay. All six samples were tested, with the low sample measured in eight replicates and the remaining five samples measured in four replicates.

### 2.6. Analytical Specificity

Analytical specificity and interference testing followed CLSI EP07 [27] guidelines by evaluating potential endogenous interferents, cross-reactive substances, common drugs, endogenous proteins, and AD-specific therapeutics. Interference and cross-reactivity testing utilized a healthy control plasma sample spiked with recombinant GFAP to a clinically relevant concentration (10 pg/mL). Potential interferents or cross-reactants were spiked into this sample and analyzed in triplicate. Analytical specificity was assessed by calculating the percentage difference between mean cross-reactant- or interferent-spiked and control sample results.

Hemoglobin interference was evaluated at concentrations up to 10 mg/mL, corresponding to severe gross hemolysis. Samples exhibiting gross hemolysis are routinely flagged and excluded from testing per standard clinical laboratory analytical acceptance criteria; therefore, this concentration represents the clinically relevant upper boundary for interference assessment.

### 2.7. Calibrator and Sample Stability

Calibrator stability was assessed per CLSI EP25 [28] guidelines under two conditions: (1) storage at 10 °C for up to 19 days (tested on days 1, 4, 6, 10, and 19) and (2) five freeze–thaw cycles. The signal representing five specific concentrations at baseline and which span the analytical measurement range was analyzed at each time point, and percent deviation from baseline was calculated for each condition. Sample stability was assessed for 5 newly collected plasma samples over a 48 h period (hours 6, 24, 48) at room temperature (20–25 °C) and over 5 freeze–thaw cycles, with percentage differences from baseline reported.

Samples were thawed overnight at 4 °C prior to testing. This controlled low-temperature thawing protocol was selected to minimize thermal denaturization and protein precipitation associated with rapid room-temperature thawing of samples stored at −80 °C. Analyte stability under these conditions was confirmed by sample stability studies demonstrating <4% deviation in GFAP concentration over 48 h at 4 °C.

### 2.8. Sample Type Comparison

For MS applications, serum has been the standard matrix [16,17]. Plasma and serum matrix interchangeability were validated utilizing matched K2 EDTA plasma and serum samples (BioIVT, Westbury, NY) from relapsing–remitting MS (RRMS)-diagnosed samples (*n* = 10, median age 53, range 41–74) and healthy controls (*n* = 13, median age 57, range 55–80). The samples were evaluated on the Dxl 9000 Access Immunoassay Analyzer (Beckman Coulter, Brea, CA, USA).

### 2.9. Method Comparison

Method comparison was performed to assess correlation between the Beckman Coulter Access GFAP (RUO) assay and the Quanterix Simoa^®^ GFAP Advantage Plus (RUO) assay (Billerica, MA, USA). Plasma samples encompassing the AMR were tested, including samples from participants with AD and healthy controls. Duplicate testing of all samples was conducted using a single reagent lot, with Simoa analysis performed by Frontage Laboratories (Exton, PA, USA). Method comparison was performed using Pearson correlation to assess association, Passing–Bablok regression to evaluate systematic bias, and Bland–Altman analysis to determine limits of agreement (LoAs).

### 2.10. Clinical Performance

Clinical performance evaluation compared GFAP levels in plasma from AD participants versus healthy controls. Data are displayed as boxplots showing median, interquartile range (IQR), and 95% confidence interval whiskers. Between-group differences were tested using the Mann–Whitney U test, with *p*-values reported with a significance threshold of 0.05.

## 3. Results

The GFAP immunoassay demonstrated analytical validity across all parameters evaluated. A summary of the analytical criteria and the results of determination for precision, sensitivity, linearity, dilution recovery, analytical specificity, and calibrator and sample stability are presented in Table 1. Performance in clinical applications was demonstrated through method comparison with the Quanterix Simoa GFAP assay and differentiation between disease and control cohorts as reported below.

**Table 1:** Summary of analytical criteria and results of determination.

Parameter	Analytical Criteria	Determination
Precision	CV < 10% across within-run, between-run, and total	Met criteria. High precision demonstrated with a CV < 10.0% across within-run, between-run, and within-lab (Table 2).
Sensitivity LLoQ Reportable Range (RR)	CV < 6.4% (k × CVr) No criteria	0.083 pg/mL 0.083–640.00 pg/mL
Linearity	<15% nonlinearity	Met criteria. Native antigen nonlinearity ranged from −3.23–8.67% (Supplementary Figure S1A). Recombinant antigen nonlinearity ranged from −1.6 to 3.6% (Supplementary Figure S1B).
Analytical Specificity	<10% difference from baseline for cross-reactants, common drugs, proteins, and lipids, and AD drugs	Met criteria for cross-reactants. Interference < 5% across substances (Table 3). Met criteria for common drugs, lipids and proteins. Interference < 7% across substances (Table 3). Met criteria for AD drugs. Interference < 2% across substances (Table 3).
Calibrator Stability	<20% difference from baseline for storage (10 °C) and freeze–thaw conditions	Met criteria for storage. Demonstrated <12% difference over 19 days (Supplementary Table S1). Met criteria for freeze–thaw. Observed <5% across 5 cycles (Supplementary Table S1).
Sample Stability	No criteria	Stable (<4% difference from baseline) over 48 h stored at room temperature and 4 °C (Supplementary Table S2). Stable (<9% difference) over 5 freeze–thaw cycles (Supplementary Table S3).

**Table 2:** Mean GFAP concentrations and coefficients of variation in endogenous and recombinant samples.

Sample ID	Mean GFAP Concentration (pg/mL)	*N*	Within-Run CV (%)	Between-Day/Run CV (%)	Within-Laboratory CV (%)
**1E**	3.7	30	2.0	0.7	3.7
**2R**	4.7	40	2.1	0.9	2.6
**3E**	6.6	40	3.2	0.0	3.3
**4R**	8.1	30	1.6	1.5	3.3
**5E**	17.9	30	1.8	1.2	7.8
**6E**	28.0	30	1.8	0.6	3.0
**7R**	94.7	35	4.6	8.3	9.5
**8R**	152.7	30	1.8	0.7	2.4

E, endogenous; R, recombinant; CV, coefficient of variation; GFAP, glial fibrillary acidic protein; ID, identifier.

### 3.1. Precision

Precision was evaluated across eight plasma samples spanning a 3.7–152.7 pg/mL concentration (Table 2). Maximum CVs were 4.6% (within-run), 8.3% (between-run), and 9.5% (within-laboratory), meeting the ≤10.0% criterion (Table 1).

### 3.2. Sensitivity

**Figure 2:**
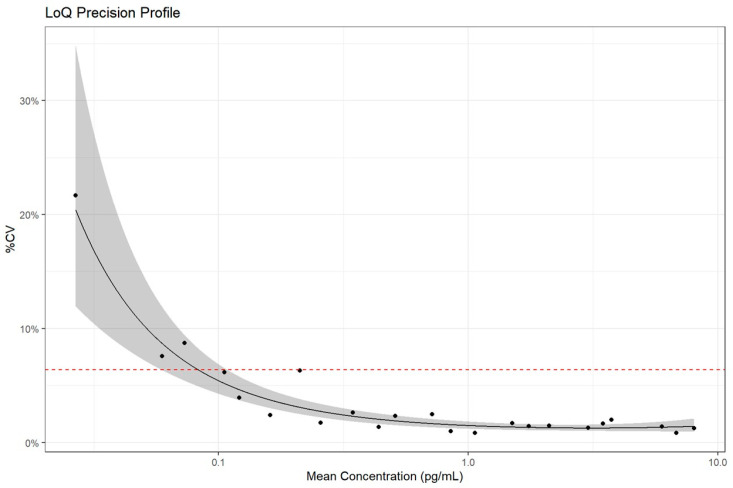
Lower limit of quantification (LLoQ) K2 EDTA plasma samples were run in duplicate and fit log–log quadratic regression to model overall precision profiles. The dashed line represents the CV threshold of 6.4%.

The lower limit of quantification (LLoQ), determined as the concentration at which the precision profile model fit crossed the CV threshold = 6.4%, was established at 0.083 pg/mL (Figure 2). The resulting AMR (reportable range) spanned 0.083–640 pg/mL; all native samples evaluated fell within this range.

Although not the primary objective of this analysis, precision was evaluated across a TBI-relevant concentration range of 23.2–1054.7 pg/mL using a high-concentration calibrator set with the upper calibrator assigned at 10,000 pg/mL (Supplementary Table S5). Within-laboratory CVs tested at the highest concentrations (822.1 pg/mL: 2.5%; 1054.7 pg/mL: 2.7%) were within the <10% acceptance criteria, demonstrating accurate and reproducible measurements at concentrations exceeding the standard AMR. Dilution recovery was assessed from a high-concentration sample (~1050 pg/mL) at 4×, 16× and 64× dilution factors, yielding recovery of 90.2–111.0% across all conditions (Supplementary Table S6).

### 3.3. Linearity

For the native antigen linearity study, nonlinearity (i.e., the difference between the observed vs. predicted value from a linear model) ranged from −3.23% to 8.67% for all concentrations observed as plotted in Supplementary Figure S1A. For the recombinant antigen linearity study, nonlinearity ranged from −1.6% to 3.6% for all concentrations observed across the AMR as plotted in Supplementary Figure S1B. This met the <15% nonlinearity criteria, reported in Table 1.

### 3.4. Analytical Specificity

The GFAP immunoassay demonstrated analytical specificity producing concentration differences between control and test samples of <5% differences across cross-reactants, <7% across common drugs, and <2% across AD drugs (Table 3); these results met the <10% difference criteria as reported in Table 1.

**Table 3:** Analytical specificity across cross-reactants.

Category	Interferent	Interferent Concentration	GFAP Control Concentration (pg/mL)	GFAP Test Concentration (pg/mL)	% Concentration Difference
Cross-reactants	Amyloid beta 1–40	10 ng/mL	12.5	12.8	2.4%
	Amyloid beta 1–42	10 ng/mL	12.5	13.0	4.0%
	Desmin	130 ng/mL	12.2	11.9	−2.5%
	Internexin	80 ng/mL	12.2	12.3	0.8%
	Keratin	10 ng/mL	12.2	12.2	0.0%
	Nestin	100 ng/mL	12.2	12.8	4.9%
	Neurofilament heavy	80 ng/mL	12.2	12.2	0.0%
	Neurofilament light	0.07 ng/mL	12.2	12.5	2.5%
	Neurofilament medium	10 ng/mL	12.2	12.1	−0.8%
	Peripherin	5 ng/mL	12.2	12.4	1.6%
	S-100-B	100 ng/mL	12.2	12.6	3.3%
	Synemin	100 ng/mL	12.2	12.2	0.0%
	Vimentin	360 ng/mL	12.2	12.4	1.6%
Common Drugs, Proteins and Lipids	Acetaminophen	0.156 mg/mL	18.1	18.1	0.0%
	Acetylsalicylic acid	0.03 mg/mL	18.1	18.0	−0.6%
	Bilirubin, conjugated	0.4 mg/mL	18.1	17.3	−4.4%
	Bilirubin, unconjugated	0.4 mg/mL	18.1	17.0	−6.1%
	Biotin	0.004 mg/mL	18.1	18.0	−0.6%
	Hemoglobin	10 mg/mL	18.1	17.6	−2.8%
	Heparin	3.3 units/mL	18.1	17.9	−1.1%
	Human gamma globulin	0.15 g/mL	18.1	17.4	−3.9%
	Human serum albumin	0.15 g/mL	18.1	18.0	−0.6%
	Ibuprofen	0.219 mg/mL	18.1	18.3	1.1%
	Intralipid	15 mg/mL	18.1	17.6	−2.8%
	Triolein	15 mg/mL	18.1	17.9	−1.1%
Alzheimer’s Drugs	Aripirazole	1800 ng/mL	18.1	18.3	1.1%
	Donepezil	300 ng/mL	18.1	17.9	−1.1%
	Galantamine	500 ng/mL	18.1	18.2	0.6%
	Memantine	450 ng/mL	18.1	18.2	0.6%
	Rivastigmine	1200 ng/mL	18.1	18.0	−0.6%

### 3.5. Calibrator and Sample Stability

Calibrator stability was confirmed with <12% deviation from baseline over 19 days of storage at 10 °C and <5% deviation over five freeze–thaw cycles (Supplementary Table S1), both meeting the criterion of <20% (Table 1). Similarly, sample stability was maintained <4% difference over 48 h at 4 °C (Supplementary Table S2) and <9% difference over five freeze–thaw cycles (Supplementary Table S3), meeting criteria as reported in (Table 1).

### 3.6. Sample Type Comparison

Plasma–serum equivalence in RRMS and healthy controls was established by Passing–Bablok regression with Pearson correlation, as well as Bland–Altman analysis. The correlation coefficient (R = 0.997), Passing–Bablok regression (y = 0.18 + 0.98x) and Bland–Altman analysis [median percentage difference 1.97%, 95% LoA (−24.94–19.39%)] confirmed robust agreement between plasma and serum (Supplementary Figure S2).

### 3.7. Method Comparison

**Figure 3:**
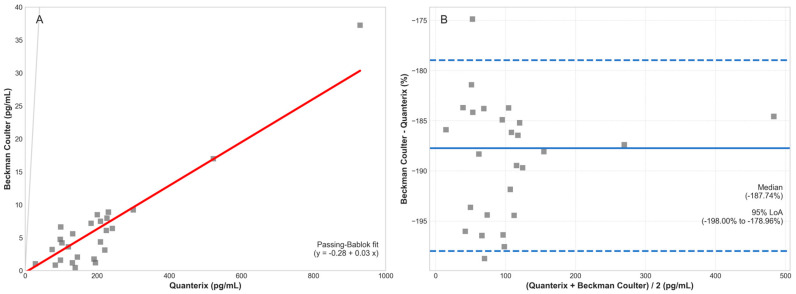
Method comparison of Quanterix and Beckman Coulter GFAP immunoassays in plasma. (**A**) Passing–Bablok regression analysis. (**B**) Bland–Altman analysis (%). Solid line indicates the median relative difference between assays, and the dashed line indicates 95% statistical limits of agreement (LoAs).

The GFAP immunoassay was highly correlated (R = 0.945) with the Quanterix Simoa GFAP RUO assay across the 26 plasma samples tested ranging from 28.4 to 929.0 pg/mL. Despite strong correlation, a systematic difference in absolute concentrations was observed between platforms, with the Passing–Bablok regression equation of y = −0.28 + 0.030x indicating the Beckman GFAP assay reports approximately 3% of Quanterix Simoa GFAP values (Figure 3A). Bland–Altman analysis (Figure 3B) confirmed systematic proportional bias between methods, with a median percentage difference of −187.7% (95% LoA −198.0% to −179.0%).

### 3.8. Clinical Performance

**Figure 4:**
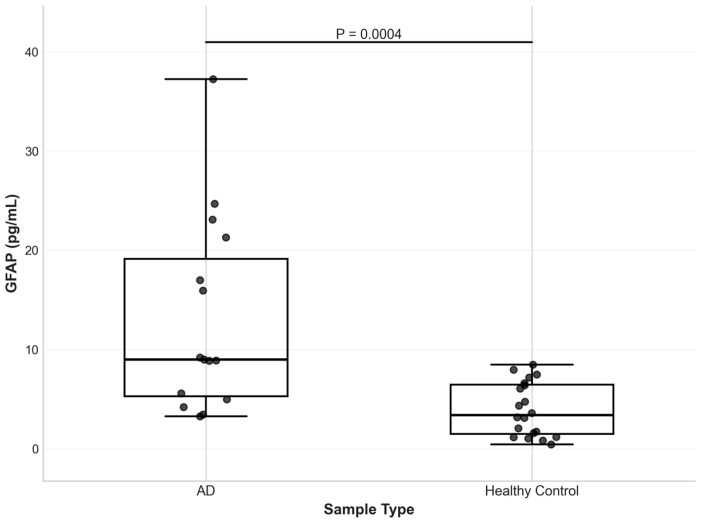
Preliminary clinical performance of Access GFAP assay in healthy controls and samples from patients with Alzheimer’s disease (AD). Lines and boxes depict median and quartiles, with minimum and maximum as whiskers.

Clinical performance was assessed by comparing plasma GFAP levels between healthy controls and disease cohorts. Plasma GFAP concentrations from healthy controls (*N* = 20, median age 65 years, range 55–70) were compared with those from AD participants (*N* = 15, median age 69 years, range 52–83) with mild-to-moderate cognitive impairment (MMSE 16–26). The AD group exhibited significantly elevated GFAP levels (median 9.0 pg/mL, IQR: 5.3–19.1 pg/mL) compared with controls (median 3.4 pg/mL, IQR: 1.5–6.5 pg/mL; *p* < 0.001) as displayed in Figure 4.

## 4. Discussion

GFAP is increasingly recognized as a diagnostic and prognostic biomarker in neurodegenerative diseases, creating the need for analytically robust assays. This study characterized the analytical and clinical relevance of the Access GFAP (RUO) immunoassay (Beckman Coulter, Brea, CA, USA) in plasma. The GFAP assay demonstrated strong analytical performance across all evaluated parameters (Table 1) with high sensitivity (LLoQ = 0.083 pg/mL) supporting measurement across the full AD spectrum, including preclinical stages. Method comparison to the Quanterix Simoa GFAP RUO assay demonstrated strong correlation (R = 0.945) but substantial systematic proportional bias (Passing–Bablok slope = 0.03; Bland–Altman median difference = −187.7%), consistent with patterns observed across other plasma-based GFAP assays [21,29]. Study observations of elevated GFAP levels in AD (median 9.0 pg/mL) compared with controls (median 3.4 pg/mL), when adjusted for platform bias, aligned with expected values from prior studies that utilized the Simoa GFAP RUO assay [30,31,32]. A comparison of RUO GFAP assays is provided in Supplementary Table S4.

Several mechanisms contribute to this systematic bias. First, as recently reviewed by Gogishvili et al. [20], the epitopes targeted by commercially available GFAP immunoassays are largely unknown or poorly characterized, and the two platforms employ different monoclonal antibody clones. Differences in epitope accessibility, antibody affinity, and recognition of native versus post-translationally modified or proteolytically fragmented GFAP can produce systematic differences in the fraction of circulating GFAP detected, independent of total analyte concentration. Second, the platforms utilize fundamentally different calibration strategies: the Quanterix Simoa assay is calibrated using proprietary reference materials, while the Access GFAP assay uses Beckman Coulter-assigned calibrators. In the absence of a commutable international reference standard for plasma GFAP, like those established for creatinine or HbA1c, each platform operates on an independent value-assignment chain, making absolute concentration agreement unlikely even when both assays accurately measure the same biological pool. Third, differences in signal detection (chemiluminescence in the Access platform versus fluorescence-based digital single-molecule counting in Simoa) may contribute to platform-specific response characteristics. Despite these differences in absolute values, the strong correlation (R = 0.945) confirms that both platforms are detecting the same biological signal, and the Access GFAP assay AD/control fold-difference (2.6-fold) is consistent with fold-differences reported using the Simoa platform [30,33], supporting biological concordance despite the lack of absolute harmonization and values should not be directly compared. Cross-platform interpretation should therefore rely on relative group differences and fold-changes rather than on absolute concentrations. These findings reinforce the need for development of a commutable plasma GFAP reference material and reference measurement procedure to enable cross-platform harmonization [21].

Future validation should include undiluted spike–recovery assessment across diverse plasma matrices to fully characterize the impact of endogenous matrix variation on assay performance. Additionally, formal evaluation of the rheumatoid factor interference and the recognition of proteolytically cleaved GFAP fragments—circulating proteoforms known to be clinically relevant in both TBI and neurodegeneration [20]—represent important areas for future analytical characterization prior to clinical implementation. We were limited by our sample size (*N* = 35), and larger clinical validation studies are needed to confirm our findings.

Multiple lines of evidence support the utility of plasma GFAP as an AD biomarker across the disease continuum, particularly in early stages [6,9,10,11,12,13,14,15]. In the Alzheimer’s and Families (ALFA) longitudinal study, plasma GFAP was detectable in cognitively unimpaired individuals with early AD pathology, with structural equation modeling revealing that GFAP mediated the relationship between soluble and insoluble Aβ and correlated with fibrillar Aβ deposition on PET [10].

Plasma GFAP also associates with tau abnormalities only in Aβ-positive individuals, though levels plateau beyond a certain tau threshold and may inversely correlate with Aβ-PET in those with severe impairment [11,12]. Supporting evidence from a larger cohort (*n* = 262) demonstrates the diagnostic value of the ACCESS GFAP RUO assay in AD, including correlation with Aβ-PET [34].

As a prognostic marker, GFAP predicts cognitive decline and cortical atrophy in AD signature regions [13]. However, GFAP is elevated with age and other forms of dementia (cognitive decline) confounding its utility as a specific AD standalone biomarker [6,9,14,15,31]. These findings position GFAP as a context-dependent indicator of astrocytic activation with potential utility in multi-marker panels for AD [6,9,33].

While this study evaluated the analytical validity and clinical performance of the Access GFAP RUO assay for AD application, the assay’s performance characteristics support broader utility across neurological conditions. We demonstrated sample type agreement between plasma and serum in relapsing–remitting MS and healthy controls, enabling potential use in diverse neurodegenerative conditions.

GFAP concentrations vary substantially by pathology: neurodegenerative diseases including AD, MS, and ALS demonstrate overlapping GFAP ranges, whereas TBI produces dramatically higher levels. For example, in TBI, plasma GFAP can increase rapidly to more than 10-fold above baseline, while AD shows more-modest but sustained elevations over years, typically 2- to 4-fold of control levels [4,19,35]. Given this wider dynamic range required for TBI applications, precision and dilution recovery analyses were performed across TBI-relevant concentrations (Supplementary Materials, Traumatic Brain Injury Supplement), demonstrating assay capability across both neurodegenerative and acute neurological injury contexts. However, as a formal hook effect assessment was not performed at TBI-relevant concentrations (e.g., up to 10,000 pg/mL), this assay has not yet been fully analytically validated for TBI. Additionally, it is important to note that TBI applications require sample dilution for concentrations exceeding the 640 pg/mL AMR. Our analysis confirmed dilution recovery to be accurate over the concentration range tested.

Dedicated clinical validation studies will be required to confirm performance in TBI, MS, and ALS populations. Additionally, a hook effect assessment at TBI-relevant concentrations will be required to confirm analytical validity for TBI. However, the ability to leverage a single high-throughput platform for GFAP across multiple clinical indications represents significant value for clinical laboratory implementation.

## Data Availability

The raw data supporting the conclusions of this article will be made available by the authors on request.

## Supplementary Materials

The following supporting information can be downloaded at https://www.mdpi.com/article/10.3390/diagnostics16132060/s1, Supplementary Figure S1. Linearity Dilution Testing. Supplementary Figure S2. Serum and EDTA Plasma Sample Type Comparison. Supplementary Table S1: Calibrator Stability. Supplementary Table S2: Sample Stability at Room Temperature. Supplementary Table S3: Sample Stability through Freeze-Thaw Cycles. Supplementary Table S4: Comparison of RUO GFAP Assays.Supplementary Table S5: Precision of the GFAP assay in plasma in TBI-relevant range. Supplementary Table S6: Dilution recovery.

## Abbreviations

The following abbreviations are used in this manuscript:

GFAP	Glial fibrillary acidic protein
RUO	Research use only
AD	Alzheimer’s disease
CV	Coefficient of variation
LLoQ	Lower limit of quantification
IQR	Interquartile range
TBI	Traumatic brain injury
CNS	Central nervous system
MS	Multiple sclerosis
ALS	Amyotrophic lateral sclerosis
CLSI	Clinical and Laboratory Standards Institute
AMR	Analytical measurement range
PET	Positron emission tomography
