# Supplementary material for: Analytical Performance and Evaluation in Clinical Cohorts of a Fully Automated Immunoassay for Plasma Glial Fibrillary Acidic Protein
Source: Diagnostics (Basel). 2026 Jul 1;16(13):2060. doi: 10.3390/diagnostics16132060 (PMC13359704; doi:10.3390/diagnostics16132060)
Supplement: Supplementary file 1 [file diagnostics-16-02060-s001.zip › diagnostics-4348662-supplementary.pdf]

Supplementary Material

Analytical Validation Supplement

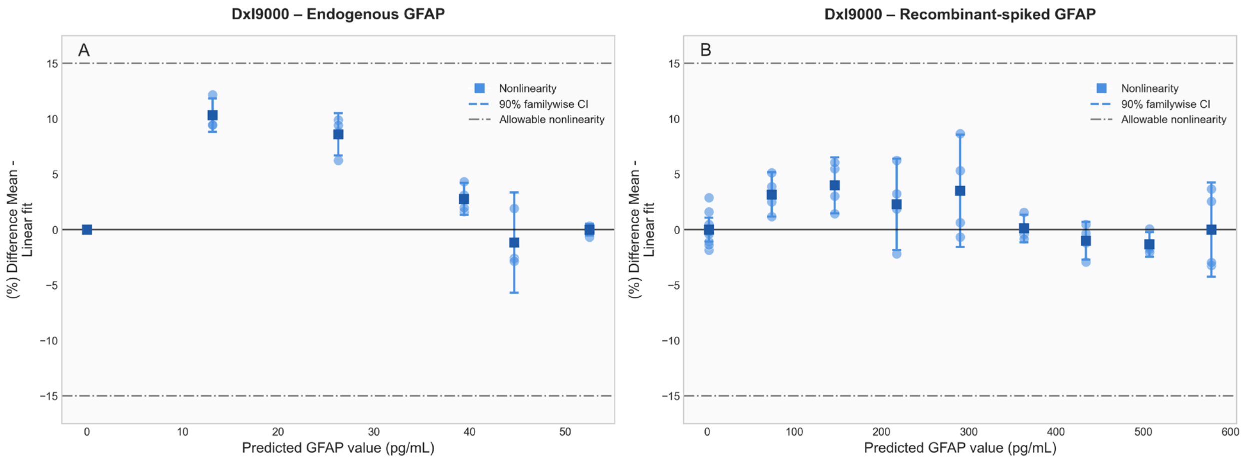

Supplementary Figure S1. Linearity Dilution Testing

A. Difference in linear fit in endogenous CSF antigen spike-in. Error bar for the predicted value of GFAP value of 0 pg/mL is not displayed due to undefined variance at zero concentration.

B. Difference in linear fit in recombinant GFAP samples.

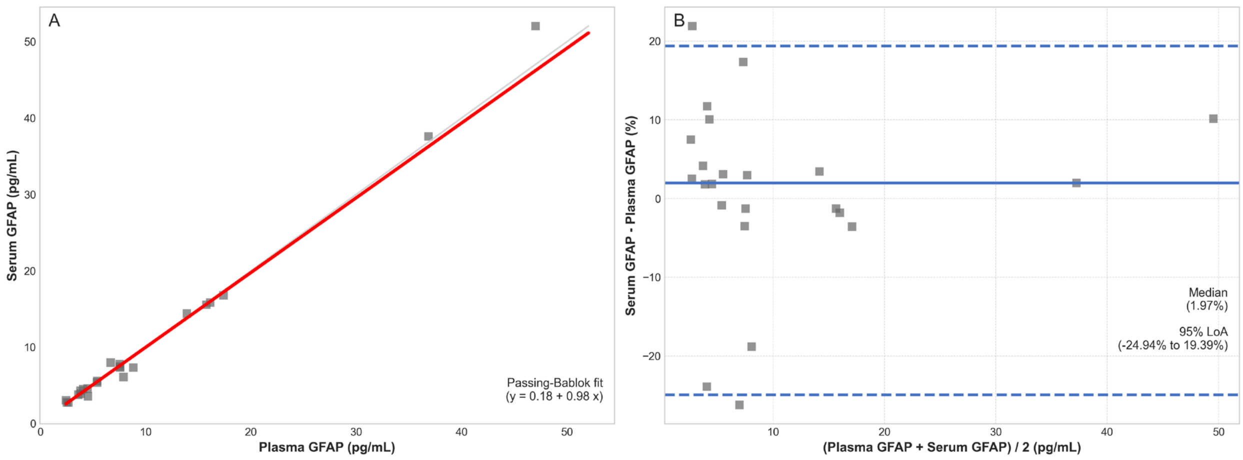

Supplementary Figure S2. Serum and EDTA Plasma Sample Type Comparison

A. Plasma-serum equivalence was established by Passing-Bablok regression (R=0.997).

B. Bland-Alman analysis confirmed agreement between plasma and serum (median percentage difference = 1.97%, LoA (95%) = -24.94% to 19.39%).

Supplementary Table S1: Calibrator Stability

Concentration at Baseline (pg/mL)	% Difference from baseline over time at 10C					
	**Day 0**	**Day 1**	**Day 4**	**Day 6**	**Day 10**	**Day 19**
1.3 pg/mL	0%	1%	5%	1%	1%	-2%
4 pg/mL	0%	4%	5%	1%	0%	0%
13 pg/mL	0%	7%	7%	1%	-1%	3%
100 pg/mL	0%	7%	9%	6%	9%	5%
300 pg/mL	0%	8%	11%	9%	7%	10%
**Concentration at Baseline (pg/mL)**	**% Difference from baseline over freeze-thaw cycle**					
	**F-T 0**	**F-T 1**	**F-T 2**	**F-T 3**	**F-T 4**	**F-T 5**
1.3 pg/mL	0.0%	2.8%	0.8%	-0.8%	1.7%	-1.6%
4 pg/mL	0.0%	0.2%	1.2%	-0.8%	2.2%	0.5%
13 pg/mL	0.0%	-3.2%	0.4%	-1.8%	1.5%	0.1%
100 pg/mL	0.0%	0.8%	-0.2%	1.0%	1.8%	-1.3%
300 pg/mL	0.0%	2.0%	1.8%	2.4%	3.1%	4.6%

Percentage (%) difference from baseline over time (1 through 19 days) at 10°C and over freeze-thaw cycles.

Supplementary Table S2: Sample Stability at Room Temperature

Dose, pg/mL	% Difference from baseline over time		
	**Hour 6**	**Hour 24**	**Hour 48**
2.33	-1.9%	-2.2%	1.2%
3.20	-1.4%	-2.7%	-1.6%
4.78	0.8%	0.9%	3.1%
8.05	-1.5%	0.5%	-1.2%
8.71	-3.4%	-1.7%	-1.5%

Supplementary Table S3: Sample Stability through Freeze-Thaw Cycles

Dose, pg/mL	% Difference from baseline over freeze-thaw cycle				
	**F-T 1**	**F-T 2**	**F-T 3**	**F-T 4**	**F-T 5**
5.35	0.4%	-2.6%	-1.9%	-1.6%	-6.6%
12.02	-1.6%	-2.9%	-4.4%	-2.2%	-8.1%
15.55	-1.2%	-0.9%	-3.1%	-4.1%	-4.8%
19.15	-0.7%	-0.7%	-4.3%	-2.2%	-5.4%
20.55	-0.6%	-2.1%	0.1%	-0.3%	-3.0%

Supplementary Table S4: Comparison of RUO GFAP Assays

Platform	Technology	Automation	LOD (plasma/serum)	LLOQ (plasma/serum)	Measuring/dynamic range	Sample types	Sample volume
**Beckman Coulter ACCESS/Dxl 9000**	Chemiluminescent immunoassay with 1-step sandwich, Lumi-Phos	Fully automated Random access	< 1 pg/ml	0.0083 pg/mL	0.083-640pg/mL* (plasma)	EDTA plasma	100 μL
**Quanterix Simoa HD-X (Advantage PLUS)**	Digital ELISA Single molecule array	Plate-based batch	~0.2 pg/mL	0.69-1.37 pg/mL	0-4,000 pg/mL (plasma) 0-40,000 pg/mL (CSF)	EDTA plasma, serum, CSF	~152 μL (total)
**Fujirebio Lumipulse**	Chemiluminescent enzyme immunoassay	Fully automated random-access	~2-4 pg/mL	~6 pg/mL	4-5,000 pg/mL	EDTA plasma, serum, CSF	~100 μL (+ 100 μL dead vol on analyzer)
**Roche Elecsys Cobas e**	Electrochemiluminescent Immunoassay	Fully automated random access	~1-3 pg/mL	~1-5 pg/mL	~1-50,000+ pg/mL (broad, on-board dilution available)	Serum, EDTA plasma, CSF	~ 50 μL

LOD=limit of detection, LLOQ=lower limit of quantification. Simoa specs from Quanterix Discovery Kit datasheets (HD-X, SR-X). Fujirebio LoQ from Nojima et al. 2025 (https://doi.org/10.1002/alz.094628) and Diagnostics 14:2520, 2024. Roche specs are based on published literature and Roche eLabDocs.

*Greater range possible (up to 10,000) with dilution.

Traumatic Brain Injury Supplement

Precision

Precision was assessed across the TBI-relevant concentration range (23.2–1054.7 pg/mL) using 3 replicates per run over 5 days. To generate doses with higher concentration samples (> 640 pg/mL), a high concentration calibrator set was used, with the top end of the calibration curve assiagned at 10,000 pg/mL. One sample (R8*) was identified as a statistical outlier; per CLSI EP05-A3, results are reported both with and without this outlier (Supplementary Table S5). Maximum CVs excluding the outlier were 3.5% (within-run), 2.2% (between-run), and 4.1% (within-laboratory). With or without the outlier, the within-laboratory CV met the < 10% criteria established.

Supplementary Table S5: Precision of the GFAP assay in plasma in TBI-relevant range

Sample ID	Mean GFAP Concentration (pg/mL)	Within-Run CV (%)	Between-Day/Run CV (%)	Within-Laboratory CV (%)
R6	23.2	3.5%	4.1%	5.4%
R7	88.1	3.8%	1.9%	4.3%
R8<br>*R8**	281.5<br>*288.3**	3.5%<br>*9.7%**	2.2%<br>*0.0%**	4.1%<br>*9.7%**
R9	623.0	1.5%	2.5%	2.9%
R10	822.1	1.2%	2.2%	2.5%
R11	1054.7	2.4%	1.4%	2.7%

Precision of recombinant (Sample ID R) GFAP in plasma; CV, coefficient of variation; GFAP, Glial Fibrillary Acidic Protein; ID, identifier; * indicates analyses of the R8 sample with the outlier

Dilution Recovery

Dilution recovery may be important for quantifying samples exceeding the upper AMR, particularly relevant for TBI applications where GFAP concentrations can reach very high levels. Dilution recovery was assessed using a high-concentration sample prepared by spiking recombinant GFAP into plasma to reach the upper calibrator range. The sample was serially diluted with wash buffer at 4×, 16×, and 64× dilution factors. To generate this dose, a high concentration calibrator set was used, with the top end of the calibration curve assigned at 10,000 pg/mL. Recovery ranged from 90% to 111% across all dilution factors (Supplementary Table S6), meeting a generally accepted criteria of 80–120%.

Supplementary Table S6: Dilution recovery

Dilution Factor		Low Sample		Washer Buffer	
	Target (pg/mL)	Measured (pg/mL)	Recovery (%)	Measured (pg/mL)	Recovery (%)
4	262.5	260.9	99.4%	291.4	111.0%
16	65.6	59.2	90.2%	64.6	98.5%
64	16.4	18.0	109.8%	15.8	96.3%
